# Validation of Ultrasound Risk Stratification Systems for Cervical Lymph Node Metastasis in Patients with Thyroid Cancer

**DOI:** 10.3390/cancers14092106

**Published:** 2022-04-23

**Authors:** Ji Ye Lee, Roh-Eul Yoo, Jung Hyo Rhim, Kyung Hoon Lee, Kyu Sung Choi, Inpyeong Hwang, Koung Mi Kang, Ji-hoon Kim

**Affiliations:** 1 Department of Radiology, Seoul National University Hospital, Seoul National University College of Medicine, Seoul 03080, Korea; peachwh@naver.com (J.Y.L.); roheul7@gmail.com (R.-E.Y.); leekyung525@gmail.com (K.H.L.); kyuchoi86@gmail.com (K.S.C.); mit3000kr@gmail.com (I.H.); we3001@gmail.com (K.M.K.); 2 Department of Radiology, Seoul Metropolitan Government Seoul National University Boramae Medical Center, Seoul 156707, Korea; wjdgy1127@gmail.com

**Keywords:** lymph nodes, risk, thyroid cancer, ultrasonography

## Abstract

**Simple Summary:**

Ultrasound (US) malignancy risk stratification systems (RSS) for cervical lymph nodes (LNs) have not been fully established in patients with thyroid cancer. In this study, we assessed the malignancy risks of each US feature and risk category from the Korean Society of Thyroid Radiology (KSThR) and the European Thyroid Association (ETA). Both systems effectively classified malignancy risks; however, 15.1% of LNs were unclassifiable in ETA RSS. Suspicious US features of hyperechogenicity, cystic change, echogenic foci, and abnormal vascularity were independently associated with metastasis. When the primary tumor characteristics were assessed, tumor multiplicity was associated with metastasis in the indeterminate LN group. We refined this system and proposed an RSS based on the KSThR system for cervical LNs in patients with thyroid cancer.

**Abstract:**

A malignancy risk stratification system (RSS) for cervical lymph nodes (LNs) has not been fully established. This study aimed to validate the current RSS for the diagnosis of cervical LN metastasis in thyroid cancer. In total, 346 LNs from 282 consecutive patients between December 2006 and June 2015 were included. We determined the malignancy risk of each ultrasound (US) feature and performed univariable and multivariable logistic regression analyses. Each risk category from the Korean Society of Thyroid Radiology (KSThR) and the European Thyroid Association (ETA) was applied to calculate malignancy risks. The effects of size, number of suspicious features, and primary tumor characteristics were analyzed to refine the current RSS. Suspicious features including echogenic foci, cystic change, hyperechogenicity, and abnormal vascularity were independently predictive of malignancy (*p* ≤ 0.045). The malignancy risks of probably benign, indeterminate, and suspicious categories were 2.2–2.5%, 26.8–29.0%, and 85.8–87.4%, respectively, according to the KSThR and ETA criteria. According to the ETA criteria, 15.1% of LNs were unclassifiable. In indeterminate LNs, multiplicity of the primary tumor was significantly associated with malignancy (odds ratio, 6.53; *p* = 0.004). We refined the KSThR system and proposed a US RSS for LNs in patients with thyroid cancer.

## 1. Introduction

Differentiated thyroid carcinoma (DTC) is characterized by relatively indolent clinical and biological characteristics. However, DTC, especially papillary thyroid carcinoma (PTC), is frequently accompanied by cervical lymph node (LN) metastasis at the time of diagnosis (approximately 30–80%) [1,2]. In these patients, residual metastatic LNs represent the most common site of persistent disease or recurrence following incomplete resection [3,4]. Therefore, the completeness of surgical resection is one of the most important determinants of patient outcome. In this regard, an accurate preoperative diagnosis of cervical LN metastasis is necessary to reduce the chance of repetitive surgery, which is associated with a higher risk of postoperative complications [4,5,6].

Ultrasonography (US) is considered the imaging modality of choice for preoperative diagnosis and localization of LN metastasis in patients with thyroid cancer [6,7,8,9]. US plays a critical role in assessing the malignancy risk of thyroid nodules and LNs, the decision to perform US-guided biopsy, and management decisions after biopsy. The accurate identification of small but overt nodal metastases is becoming increasingly important, as active surveillance is considered an important alternative to immediate surgery in patients with papillary thyroid microcarcinoma [10].

To this end, two international societies, the European Thyroid Association (ETA) [11] and the Korean Society of Thyroid Radiology (KSThR) [12,13], proposed US-based risk stratification systems (RSSs) for cervical LNs to provide optimized management recommendations for imaging-detected LNs. Although they share similar three-tiered classification systems of “(probably) benign,” “indeterminate,” and “suspicious” for risk stratification, these classifications show differences in the details of US lexicons and categories. Despite the importance of these guidelines, they have not been fully validated, and some areas of uncertainty remain, including the malignancy risks in each lexicon and category.

With the development of guidelines and their widespread use, the role of US in assessing LNs has been further emphasized, and this background necessitates the validation of these US lexicons and RSSs. Therefore, this study aimed to determine the predictive value of suspicious US features and RSSs provided by the KSThR and ETA guidelines and to propose an RSS for LNs in preoperative patients with thyroid cancer [11,12,13].

## 2. Results

### 2.1. Baseline Characteristics of the Study Population

Of the 346 LNs, 155 (44.8%) and 191 (55.2%) were benign and malignant, respectively. In total, 339 LNs were from PTC, one from follicular carcinoma, three from medullary carcinoma, and three from anaplastic thyroid carcinoma. The mean long diameter (LD) of the index tumor was 11.8 (range, 2.0–52.0) mm. The mean short diameter (SD) and LD of the LNs were 6.0 (range, 2.2–29.1) mm and 10.3 (range, 3.2–49.7) mm, respectively. The demographic data based on each diagnostic criterion for all LN are provided in Table 1. The LD (*p* = 0.004) and SD (*p* < 0.001) of malignant LNs were significantly larger than those of benign LNs. The nodal stations of malignant LNs were significantly different from those of benign LNs, in which malignant LNs were less frequently observed in levels II and V and more frequently observed in level VI than benign LNs (*p* = 0.001).

### 2.2. Malignancy Risk Based on Each Ultrasound (US) Feature

Table 2 shows the differences between the US features of the malignant and benign LNs. All US features, except nonparallel orientation (*p* = 0.268), were significantly more frequently observed in malignant LNs than in benign LNs (*p* ≤ 0.005). Table 2 summarizes the malignancy risk and associations between the US features and malignant LNs. All suspicious US features, including echogenic foci (EF) (both punctate EF and large EF), hyperechogenicity, cystic change, and abnormal vascularity showed a high malignancy risk greater than 84%. The eccentric hilum showed the lowest malignancy risk (14.3%), followed by the loss of the hilum (68.0%). In univariable logistic regression analysis, all features, except nonparallel orientation, showed a significant association with malignancy. However, the presence of EF (odds ratio [OR], 2.6; 95% confidence interval [CI], 1.01–7.48; *p* = 0.045), hyperechogenicity (OR, 11.85; 95% CI, 4.4–31.9; *p* < 0.001), cystic change (OR, 22.91; 95% CI, 2.78–189.1, *p* = 0.004), and abnormal vascularity (OR, 1.99; 95% CI, 1.15–3.44; *p* = 0.014) were the only features independently associated with malignancy.

### 2.3. Malignancy Risk According to US Classification

Table 3 demonstrates the malignancy risks of LNs stratified by KSThR and ETA RSSs. The overall malignancy rates in the probably benign, indeterminate, and suspicious KSThR categories were 2.5% (95% CI, 0.3–9.0), 29.0% (95% CI, 17.7–44.8), and 85.8% (95% CI, 73.3–99.7), respectively. The malignancy risk was significantly different between each LN category of KSThR (*p* < 0.001, respectively).

According to the ETA criteria, the categories of normal, indeterminate, and suspicious for malignancy showed malignancy risks of 2.2% (95% CI, 0.1–12.1), 26.8% (95% CI, 6.5–29.7), and 87.4% (95% CI, 74.7–101.8), respectively, which were similar to the KSThR categories. When the LNs were categorized according to the ETA criteria, 53 (15.3%) LNs were not classified into any category. Unclassified LNs with normal hilum showed (1) slightly higher but similar malignancy risk as the normal LN category, while unclassified LNs with absent hilum showed (2) a similar malignancy risk as the indeterminate LN category. These unclassified LNs could be further stratified into two categories according to their malignancy risks: (1) LNs with normal hilum and either round shape or increased size (malignancy risk, 0–4.5%) and (2) LNs with absent hilum, oval shape, normal size, and no increased vascularity (malignancy risk, 22.6%).

### 2.4. Malignancy Risk of Suspicious Lymph Nodes (LNs) According to Nodal Size and Number of Suspicious Features

Table 4 shows the malignancy risk of suspicious LNs according to the size and number of suspicious US features. The malignancy risk of suspicious LNs in each size threshold group was similar among different size thresholds. The malignancy risk of LNs smaller than 3 mm in SD was similar to that of larger LNs. Despite no statistically significant difference, the risk of malignancy tended to be higher with an increasing number of suspicious features. Supplementary Table S1 shows the frequency of each suspicious feature according to the number of suspicious US features.

### 2.5. Association of Nodal Size (Diameter), Shape, and Primary Tumor Characteristics with Malignancy in the LN Groups

Table 5, Supplementary Tables S2 and S3 show the association of primary and nodal characteristics with malignancy in indeterminate, suspicious, and probably benign LNs, respectively. In the suspicious and probably benign LN groups, the primary tumor and LN characteristics were not significantly associated with malignancy. In indeterminate LNs, only tumor multiplicity was significantly and independently associated with malignancy in indeterminate LNs (OR, 7.4; 95% CI, 2.0–30.4; *p* = 0.003). 

### 2.6. Suggested Risk Stratification System of Cervical LNs in Patients with Thyroid Cancer

**Figure 1:**
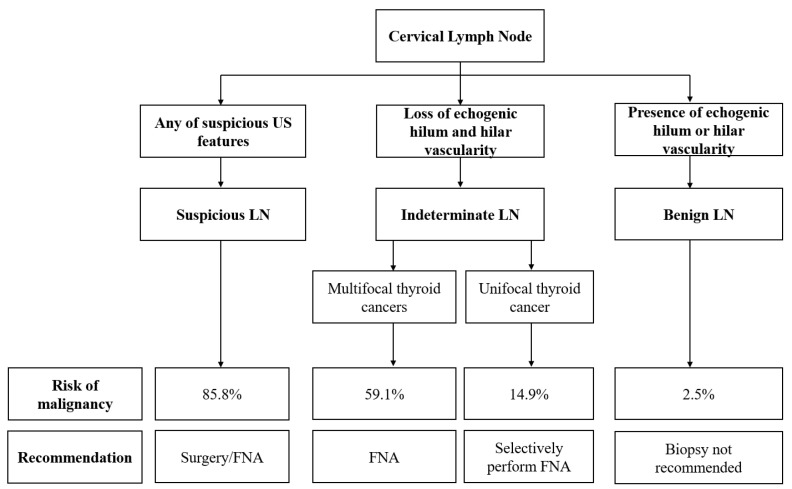
Diagram showing the algorithm for the diagnosis and management of cervical LNs in patients with thyroid cancer. LN, lymph node; FNA, fine-needle aspiration.

The malignancy risk of LNs could be stratified into three categories of KSThR according to US patterns by primarily detecting any of the four suspicious US features (EF, cystic change, hyperechogenicity, and abnormal vascularity) (Figure 1). LNs with any of these suspicious features can be categorized as suspicious, with a high malignancy risk of 85.8% (range, 72.7–100.0%). Given the high malignancy rates in tiny suspicious LNs (<3 mm SD), biopsy could be considered for suspicious LNs, regardless of their size, shape, and number of suspicious features in the preoperative setting. Fine-needle aspiration (FNA) should be selectively performed for indeterminate LNs when multiple cancers are suspected, rather than for round or enlarged LNs.

LNs with preserved hilum (regardless of eccentricity) and no suspicious features can be categorized as benign, with a significantly low malignancy risk (2.5%). Owing to the low probability of malignancy, biopsy is not routinely indicated for probably benign LNs.

## 3. Discussion

We confirmed that suspicious US features of EF, cystic change, hyperechogenicity, and abnormal vascularity were independent predictors of LN metastasis. On the contrary, features of round shape and nodal size, which have been considered suggestive of LN metastasis in several guidelines [6,11,14,15], did not predict metastasis. Different from thyroid nodules, which stratify the malignancy risk by the combination of US features [16], the estimation of malignancy risk of an LN can be determined to be high when the LN shows any of the four suspicious US features. In indeterminate LNs, tumor multifocality tended to be associated with malignancy but not with nodal US features. Based on these results, we suggest a practical RSS for cervical LNs in patients with thyroid cancer based on the KSThR LN RSS criteria.

Increased size (either LD or SD) has been reported to be a useful feature in diagnosing malignant LNs [6,11,12,14,15,17,18]. A previous study suggested performing FNA if the detected LN was >10 mm in LD or >5 mm in SD [14], whereas the practice guideline from ETA suggested performing FNA if the LN was >8 mm SD in level II and >5 mm SD in level III or IV [14]. However, in the present study, nodal size showed associations with malignancy only in the univariate analysis. Different from other head and neck malignancies, clinically significant metastatic lesions from thyroid cancer often present as small lesions in the neck [19,20]. This could be explained by the fact that PTC has a strong propensity for early LN metastasis. In addition, US (the primary imaging modality in thyroid cancer) has a superior spatial resolution for detecting pathological features in small LNs to computed tomography or magnetic resonance imaging, which is mainly used in other malignancies (which can be skipped in cross-sectional imaging) [6,7,8,21,22], especially in the lateral compartment [12,23,24,25]. In contrast to microscopic LN metastasis (<2 mm), which has little clinical significance and does not warrant aggressive surgical intervention after total thyroidectomy and radioactive iodine therapy [6,26], macroscopic metastasis has been found to be an independent prognostic factor for recurrence [27,28]. In this regard, preoperative detection and resection of small but sonographically sizable suspicious LNs may be beneficial in patients with thyroid cancer. The results of our study highlight that it is reasonable to consider the presence of suspicious features in the first place, rather than to focus on the size, for risk stratification of cervical LNs.

Loss of the nodal hilum was considered a suspicious feature of metastasis in several previous studies and guidelines [6,15,17,22,29,30]. However, loss of the nodal hilum is known for its poor specificity in diagnosing metastatic LNs [11,14]. The results of our study are in line with previous observations [11,31] that loss of nodal hilum is not an independent predictor in multivariable analysis. Similarly, a small long diameter/short diameter (L/S) ratio with a cutoff of 1.5 or 2.0 has been traditionally used as an imaging parameter to reflect the round nodal shape in malignant cervical LNs [6,11,14,32,33]. However, the round shape of LNs was not an independent predictor of malignancy in our study. These results imply that a round shape or loss of nodal hilum may be an imaging feature found along with other suspicious US features. We suggest that LNs with only a round shape or loss of hilum on US should not be categorized as suspicious.

Considering the lack of discriminative power of nodal size and shape in indeterminate LNs [32], we further focused on primary tumor characteristics, including tumor size, gross extrathyroidal extension (ETE), bilaterality, and multifocality on US. In this study, the multifocality of the primary tumor was identified as a significant and independent predictor of malignancy for indeterminate LNs, and the malignancy risk of indeterminate LNs with multifocal tumors was significantly higher than that of unifocal tumors. Multifocality is hypothesized to arise through the intraglandular spread of tumor cells from a primary focus [34] or from each independent focus [35,36]. Previous studies have suggested that tumor multifocality is significantly associated with tumor metastasis in the neck [37], as well as poorer oncologic outcomes (recurrence and death) [38]. Based on the results of our study, we propose that FNA could be actively performed for indeterminate LNs accompanied by multifocal disease, given the high incidence rate of multifocality (18–87%) in PTC [34] and its negative prognostic impact [38].

We validated that the RSS for LNs provided by the KSThR and ETA effectively stratified the malignancy risk of cervical LNs. In our study, a non-negligible percentage of LNs was unclassifiable, according to the ETA criteria; however, the malignancy risk of these LNs could be further stratified according to the presence of hilum, regardless of their shape and size. In light of these observations, we developed a practical algorithm for the risk stratification of cervical LNs in patients with thyroid cancer by adding refinements to the preexisting KSThR RSS for cervical LNs. As benign LNs on US probably have a significantly low malignancy risk (2.5%), FNA is not recommended. This is consistent with previous guidelines [11,39]. In the preoperative setting, US-depicted suspicious LNs should be considered for FNA, regardless of their size and the number of suspicious US features, because the malignancy risk of small suspicious LNs is not different from that of large suspicious LNs. FNA should be considered for indeterminate LNs accompanied by multifocal cancers because their malignancy risk is high (59.1%). FNA should be selectively performed for indeterminate LNs with unifocal cancers, and considerations should be based on the surgical strategy or overall tumor burden, rather than nodal size or shape.

Our study compared two currently available RSSs, with a suggestion of refinement according to nodal and primary tumor characteristics. The results of our study may potentially help in improving each system and providing a basis for international standardization. 

One of the limitations of the present study was its retrospective design. An inevitable selection bias for selecting LNs for US-guided biopsy may have existed. US scanning and biopsy were performed by different operators using various US machines; therefore, the determination of US categorization and biopsy may have been influenced by operator experience. In addition, the retrospective assessment of static US images has an inherent limitation to the accuracy of US interpretation. Moreover, the malignancy risk in the overall LNs and probably benign and indeterminate LN groups could have been overestimated because FNA was performed only in limited cases for these LNs. Lastly, given that the reference standard was determined by cytology or histopathology via core needle biopsy (CNB), microscopic metastases could have been overlooked. 

## 4. Materials and Methods

### 4.1. Study Population

The Institutional Review Board of Seoul National University Hospital approved this retrospective study, and the requirement for informed consent was waived owing to the retrospective nature of this study. A radiology report database search discovered 55,276 patients who underwent FNA or CNB for neck lesions at our institution between December 2006 and June 2015. The inclusion criteria accepted patients with primary thyroid cancer who underwent either FNA or CNB for cervical LNs as part of their preoperative evaluation. The exclusion criteria were as follows: (1) biopsy at sites other than LNs (*n* = 47,427), (2) a history of other malignancies (*n* = 6768), and (3) a history of previous surgery for thyroid cancer (*n* = 793), or non-diagnostic biopsy results (*n* = 8).

**Figure 2:**
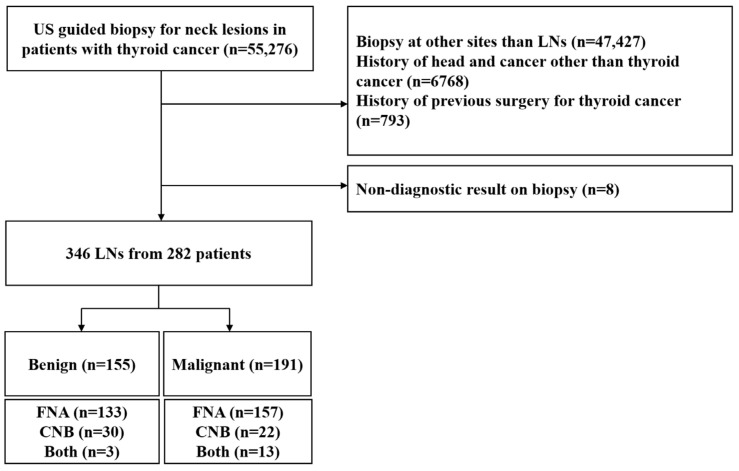
Flowchart of the study group. US, ultrasonography; LN, lymph node; FNA, fine-needle aspiration; CNB, core needle biopsy.

As a result, 346 LNs in 282 consecutive patients with thyroid cancer (72 men and 210 women; mean age, 47.9 years; age range, 18–82 years) with a final diagnosis were included in this study (Figure 2). These populations were previously analyzed, and the data were published in an article that focused on the malignancy risk of indeterminate LNs based on the 2016 KSThR classification [32].

### 4.2. US Imaging and US-Guided Biopsy

All US images were obtained by board-certified neuroradiologists using linear transducers (7.5–15.0 MHz). Grayscale and color Doppler images were examined before biopsy. US-guided biopsies were primarily conducted for indeterminate or suspicious LNs at the operator’s discretion and for benign-looking LNs upon the request of the attending physician. FNA with thyroglobulin (FNA-Tg) measurement was performed concurrently when the primary cancer was suspected to be differentiated thyroid carcinoma. One to three needle passes with a 23-gauge needle were made during FNA under continuous US guidance, and direct smears were prepared immediately by the conventional method. The remaining aspirated samples were rinsed with 1 mL of isotonic saline and submitted for FNA-Tg.

### 4.3. Reference Standard

For each LN, the final diagnosis was determined using cytology or histopathology on a node-by-node basis. Cytology or histopathology was used as the reference standard for solid LNs. For cystic LNs with insufficient cytology results (*n* = 18), an FNA-Tg cutoff of 8.3 ng/mL [39] was adapted to distinguish malignant from benign LNs. We adapted the FNA-Tg levels only in cystic LNs because elevated Tg levels could lead to false-positive diagnoses in benign ectopic thyroid tissue [40,41]. Tg was assayed using an immunoradiometric assay (RIA Tg-plus; BRAHMS GmbH, Hennigsdorf, Germany). The analytical sensitivity read at the optimal curve was 0.08 ng/mL, and the functional assay sensitivity (20% interassay coefficient of variation) was 0.2 ng/mL. All of these non-diagnostic cystic LNs were proved to be metastatic compartments based on level-by-level correlation of surgical pathologic results. In this study, the biopsy results were used as the reference standard because level-by-level correlation based on surgical pathology has limitations in that the LN identified in the operative field could not be directly correlated with the image-denoted LN [32].

### 4.4. Image Analysis

Preoperative categorization of the primary tumor and cervical LNs was performed by inspecting the recorded US images. All US images were independently analyzed by two experienced neuroradiologists (R-E. Yoo and J-h. Kim, with 9 and 20 years of experience, respectively), and discrepant cases were determined by the consensus of two reviewers.

LN laterality (ipsilateral or contralateral) was assessed with respect to the largest index tumor. The SD and LD were measured in the most representative longitudinal nodal plane, and the long-to-short diameter (L/S) ratio was calculated.

EF in the LNs were defined as focal regions that were evidently hyperechoic relative to the rest of the LN cortex and medulla. They were categorized as punctate EF (EF ≤ 1 mm within the solid component) and large EF (EF > 1 mm). Cystic change was defined as an anechoic portion within the LN. LN echogenicity was classified as hyperechoic, isoechoic, or hypoechoic using the anterior neck muscles as the reference standard. The presence or absence of an echogenic hilum within LNs was evaluated. Vascular LN configurations were categorized into three patterns (none, hilar pattern, and peripheral or diffuse) using color Doppler images [11,12].

Additional features of irregular nodal margin, nonparallel orientation (anteroposterior diameter > transverse diameter on the transverse plane) [11], eccentric cortical thickening (in LNs with preserved hilum or hilar vascularity) [18,42], round shape (L/S ratio < 1.5 and 2.0), and the presence and shape (normal vs. eccentric) of the hilum were also evaluated [11].

The size, bilaterality, multiplicity, and ETE of primary thyroid cancers and diffuse thyroid disease on US were also evaluated. Bilaterality was defined as the observation of PTCs in both thyroid lobes, and multiplicity was defined as two or more tumor foci in the thyroid gland [37]. The US criteria for gross ETE were as follows: replacement of the strap muscle, protrusion of thyroid cancer into the tracheoesophageal groove beyond the expected margin of the normal thyroid gland, and obtuse angle with the trachea [12,43]. If multiple malignant lesions were present, ETE was considered present if any of these tumors demonstrated ETE [21]. Diffuse thyroid disease was considered present when US showed diffuse enlargement or atrophy of the thyroid gland, heterogeneous echotexture, or hypoechogenicity [44].

### 4.5. Risk Stratification of Cervical LNs

Based on US findings, all LNs were categorized according to the RSSs [11,12]. According to the KSThR criteria, LNs were categorized as probably benign, indeterminate, or suspicious [12]. LNs were interpreted as suspicious if any one of the following features was present: (1) EF, (2) cystic change, (3) hyperechogenicity, or (4) peripheral or diffuse chaotic color Doppler pattern. Probably benign LNs were defined by the presence of either an echogenic hilum or hilar vascularity in the absence of any suspicious finding. LNs with no US probably benign or suspicious LN imaging features (exhibiting neither echogenic hilum nor hilar vascularity in the absence of any other suspicious finding) were defined as indeterminate.

In addition, LNs were categorized as normal, indeterminate, or suspicious for malignancy according to the ETA guidelines [11]. LNs with microcalcifications, partially cystic appearance, peripheral of diffusely increased vascularization, and hyperechoic tissue resembling the thyroid were categorized as “suspicious for malignancy.” “Normal LNs” were defined as those with preserved hilum, ovoid shape, and normal size, absent or hilar vascularization, and with no other suspicious signs. “Indeterminate” LNs were defined as LNs with an absent hilum and at least one of the following characteristics: round shape, increased short axis ≥ 8 mm in level II and ≥5 mm in levels III and IV, and increased central vascularization. LNs that did not meet any category were classified as the “unclassified” group.

### 4.6. Statistical Analyses

The chi-squared test or Fisher’s exact test were used to delineate the US features significantly associated with malignancy. After univariable analysis, stepwise multivariable logistic regression analysis was performed to determine independent US predictors among US features that were statistically significant in univariable analysis (*p* < 0.2). The malignancy risk for each category in the KSThR and ETA RSSs was calculated.

For suspicious LNs, the malignancy risks were calculated according to their size and the number of suspicious US features. Associations with primary tumor characteristics (tumor location, size, gross ETE, multiplicity, and bilaterality), along with LN characteristics (laterality, shape, and diameter) were analyzed together by univariable and multivariable logistic regression analyses in US-classified subgroups of KSThR. Multivariable logistic regression analysis was performed using a stepwise method of the factors that were statistically significant in the univariable analysis (*p* < 0.2). All statistical analyses were performed using the MedCalc software, version 11.1.1.0 (MedCalc, Mariakerke, Belgium). Statistical significance was set at *p* value < 0.05.

## 5. Conclusions

In conclusion, the RSSs from both the ETA and KSThR could effectively stratify the malignancy risks of LNs according to the presence of suspicious US features, although there were some unclassified LNs in the ETA. LNs with any suspicious US features should be regarded as metastatic and should be considered for FNA, regardless of their size in the preoperative setting. The multiplicity of the primary tumor may be helpful in determining the indication for biopsy of indeterminate LNs. We propose an algorithm based on the US features of the LN and the primary tumor for the diagnosis of metastatic LNs. The proposed algorithm will be useful for risk stratification and management decisions of cervical LNs in patients with thyroid cancer.

## Figures and Tables

**Table 1:** Demographic and clinical characteristics of patients and lymph nodes (LN).

Parameter	Benign	Malignant	*p*
No. of patients	127	155	-
No. of female patients, *N* (%)	95 (74.8%)	115 (74.2%)	0.951
Age at diagnosis (years)	45.7 ± 12.0	47.5 ± 15.0	0.209
No. of LNs	155	191	-
Method of diagnosis			0.098
FNA	133 (85.8%)	157 (82.2%)	
CNB	30 (19.4%)	22 (11.6%)	
Both	3 (1.9%)	13 (6.8%)	
Mean maximal size of largest primary tumor	9.7 ± 6.5	11.7 ± 7.9	0.214
Mean maximal size of LN	9.4 ± 4.4	11.1 ± 6.7	0.004
Mean SD of LN	4.6 ± 2.0	7.1 ± 4.3	<0.001
Laterality respect to the largest primary tumor			0.066
Ipsilateral	125 (80.6%)	167 (88.4%)	
Contralateral	30 (19.4%)	22 (11.6%)	
Location			0.001
Level I	2 (1.3%)	0 (0.0%)	
Level II	30 (19.4%)	17 (8.9%)	
Level III	41 (26.5%)	54 (28.3%)	
Level IV	61 (39.4%)	85 (44.5%)	
Level V	7 (4.5%)	2 (1.0%)	
Level VI	8 (5.1%)	27 (14.1%)	
Supraclavicular fossa	6 (3.9%)	6 (3.1%)	

LN, lymph node; FNA, fine-needle aspiration; CNB, core needle biopsy; SD, short diameter.

**Table 2:** Malignancy risk of US features and their association with malignancy.

	Malignancy Risks			Univariable *		Multivariable *	
US Features	All (*N*, %)	No. of Malignant LNs (%)	Malignancy Risk (%)	Crude OR (95% CI)	*p*	Adjusted OR (95% CI)	*p*
Any echogenic foci	133 (38.4)	117 (61.3)	88.0	12.7 (7.0, 22.9)	<0.001	2.6 (1.0, 7.5)	0.045
Punctate echogenic foci	122 (35.3)	110 (57.6)	90.2	15.0 (7.8, 28.8)	<0.001		
Large echogenic foci	25 (7.2)	21 (11.0)	84.0	4.4 (1.5, 13.2)	0.008		
Hyperechogenicity	148 (42.8)	133 (69.6)	89.8	18.0 (10.3, 35.0)	<0.001	11.9 (4.4, 31.9)	<0.001
Cystic change	62 (17.9)	60 (31.4)	96.8	33.1 (7.9, 138.1)	<0.001	22.9 (2.8, 189.1)	0.004
Abnormal vascularity	86 (24.9)	77 (40.3)	87.1	2.6 (1.8, 3.7)	<0.001	2.0 (1.2, 3.4)	0.014
Loss of hilum	259 (74.9)	175 (91.6)	68.0	7.6 (0.9, 12.5)	<0.001		
Eccentric hilum **	21 (6.1)	3 (1.6)	14.3				
Round shape (L/S ratio < 2.0)	229 (66.2)	156 (81.7)	68.6	4.5 (2.8, 7.3)	<0.001		
Round shape (L/S ratio < 1.5)	106 (30.6)	82 (42.9)	77.4	3.8 (2.3, 6.5)	<0.001	2.1 (0.9, 5.0)	0.107
LD > 10.7 mm ***	108 (31.2)	74 (38.7)	71.3	2.5 (1.6, 4.1)	<0.001		
SD > 5.4 mm ***	147 (42.5)	113 (59.2)	76.9	5.4 (3.3, 8.7)	<0.001		
Nonparallel	12 (3.5)	9 (4.7)	75.0	2.4 (0.6, 9.0)	0.198		
Irregular shape	36 (10.4)	33 (17.3)	91.7	10.1 (3.0, 33.5)	<0.001		

Note—US, ultrasound; OR, odds ratio; LN, lymph node; L/S ratio, long to short diameter ratio; LD, long diameter; SD, short diameter. * Binary logistic regression analysis. ** In LNs with hilum present, *n* = 87. *** Calculated based on the Youden index in area under the receiver operating characteristics curve.

**Table 3:** Demographic and clinical characteristics of patients and lymph nodes (LN).

Classification	All, *n* (%)	Benign, *n* (%)	Malignant, *n* (%)	Malignancy Risk (%)
**KSThR**				
Probably benign	80 (23.1%)	78 (50.3%)	2 (1.0%)	2.5
Indeterminate	69 (19.9%)	49 (31.6%)	20 (10.5%)	29.0
Suspicious	197 (56.9%)	28 (18.1%)	169 (88.5%)	85.8
**ETA**				
Normal	46 (13.3%)	45 (29.0%)	1 (0.5%)	2.2
Unclassified	53 (15.3%)	45 (29.0%)	8 (4.2%)	15.1
(1) Normal hilum + round shape	5 (1.4%)	5 (3.2%)	0 (0%)	0%
(2) Normal hilum + increased size	17 (4.9%)	16 (10.3%)	1 (0.5%)	5.9%
(3) Absent hilum + oval shape + normal size, no central vascularity	31 (9.0%)	24 (15.5%)	7 (3.7%)	22.6%
Indeterminate	56 (16.2%)	41 (26.5%)	15 (7.9%)	26.8
Suspicious for malignancy	191 (55.2%)	24 (15.5%)	167 (87.4%)	87.4

Note—KSThR, Korea Society of Thyroid Radiology; ETA, European Thyroid Association. Comparison of malignancy risks. KSThR: Benign vs. indeterminate, *p* < 0.001, indeterminate vs. suspicious, *p* < 0.001. ETA: normal vs. unclassified all, *p* = 0.04; normal vs. unclassified (1) + (2), *p* = 0.647; normal vs. unclassified (3), *p* = 0.009; indeterminate vs. unclassified (3), *p* = 0.730; unclassified all vs. indeterminate, *p* = 0.192; indeterminate vs. suspicious for malignancy, *p* < 0.001

**Table 4:** Malignancy risk of US suspicious LNs according to size thresholds and number of suspicious US features.

	Malignant LNs, *N* (%)	All Suspicious LNs, *N* (%)	Malignancy Risk (%)
**Size thresholds**			
SD < 3 mm	8 (4.7)	11 (5.8)	72.7
3≤ SD < 5 mm	42 (24.9)	54 (28.3)	77.8
5≤ SD < 7 mm	49 (29.0)	57 (29.8)	86.0
7≤ SD <10 mm	38 (22.5)	43 (22.5)	88.4
SD ≥ 10 mm	32 (18.9)	32 (16.8)	100.0
All	169 (100.0)	197 (100.0)	85.8
**Number of suspicious US features**			
None	22	149	14.8
1	21 (14.2)	28 (11.1)	75.0
2	72 (45.2)	89 (37.7)	80.9
3	54 (29.4)	58 (28.3)	93.1
4	22 (11.2)	22 (11.5)	100.0
Any suspicious feature	169 (100.0)	197 (100.0)	85.8

Note—SD, short diameter; US, ultrasound. Size 3 vs. 3–5 mm, *p* = 0.895; 3–5 vs. 5–7 mm, *p* = 0.637; 7–10 vs. >10 mm, *p* = 0.606; <3 vs. >10 mm, *p* = 0.432. Number of suspicious features: 1 vs., 2, *p* = 0.779; 2 vs. 3, *p* = 0.435; 3 vs. 4, *p* = 0.766; 1 vs. 4, *p* = 0.349.

**Table 5:** Association of nodal size, shape parameters, and primary tumor characteristics with malignant LNs in US indeterminate LNs.

US Features	Univariable		Multivariable	
	Crude OR (95% CI)	*p*	Adjusted OR (95% CI)	*p*
Diffuse thyroid disease	1.03 (0.3, 3.3)	0.964		
Maximal diameter of largest tumor	0.99 (0.9, 1.1)	0.843		
Gross ETE of largest tumor	1.9 × 10^−9^	1.9 × 10^−9^		
Multiplicity of tumor	8.3 (2.7, 28.2)	<0.001	7.4 (2.0, 30.4)	0.003
Bilaterality of tumor	5.1 (1.1, 27.4)	0.039	1.3 (0.2, 8.3)	0.802
Laterality of LN ^a^	1.6 (0.2, 10.6)	0.626		
LD of LN	0.83 (0.6, 1.2)	0.18	0.8 (0.6, 1.1)	0.259
SD of LN	0.76 (0.5, 1.2)	0.842		
L/S ratio of LN	0.67 (0.2, 2.0)	0.471		
Round shape (L/S < 2.0)	1.5 (0.5, 4.5)	0.451	-	-
Round shape (L/S < 1.5)	1.0 (0.3, 3.4)	0.964		

Note—US, ultrasound; OR, odds ratio; ETE, extrathyroidal extension; LN, lymph node; SD, short diameter; LD, long diameter; L/S ratio, long to short diameter ratio. ^a^ Contralateral location of the LN with respect to the primary tumor

## Data Availability

The datasets analyzed in this study are available from the corresponding author upon reasonable request.

## Supplementary Materials

The following supporting information can be downloaded from https://www.mdpi.com/article/10.3390/cancers14092106/s1: Table S1, Association of nodal size, shape parameters, and primary tumor characteristics with malignant LNs in US suspicious LNs; Table S2, Association of nodal size, shape parameters, and primary tumor characteristics with malignant LNs in US probably benign LNs; Table S3: Association of nodal size, shape parameters and primary tumor characteristics with malignant LNs in US probably benign LNs.
