# Supplementary material for: Validation of Ultrasound Risk Stratification Systems for Cervical Lymph Node Metastasis in Patients with Thyroid Cancer
Source: Cancers (Basel). 2022 Apr 23;14(9):2106. doi: 10.3390/cancers14092106 (PMC9105025; doi:10.3390/cancers14092106)
Supplement: Supplementary file 1 [file cancers-14-02106-s001.zip › cancers-1671645-supplementary.pdf]

Supplementary Table S1. Frequencies of suspicious US features according to numbers of suspicious US features.

Number of Suspicious US Features	Number of Suspicious LNs	Hyperechogenicity	Cystic Change	Echogenic foci	Abnormal vascularity
1	28	25 (89.3%)	1 (3.6%)	2 (7.1%)	0 (0.0%)
2	89	55 (61.8%)	13 (14.6%)	58 (65.2%)	21 (23.6%)
3	58	46 (79.3%)	24 (41.4%)	51 (87.9%)	43 (74.1%)
4	22	22 (100.0%)	22 (100.0%)	22 (100.0%)	22 (100.0%)

Supplementary Table S2. Association of nodal size, shape parameters and primary tumor characteristics with malignant LNs in US suspicious LNs.

US Features	Univariable		Multivariable	
	**Crude OR (95% CI)**	***p***	**Adjusted OR (95% CI)**	***p***
Diffuse thyroid disease	1.05 (0.4, 2.5)	0.913	1.5 (0.6, 4.3)	0.353
Maximal diameter of largest tumor	1.03 (0.9, 1.07)	0.237	1.03 (0.99, 1.1)	0.143
Gross ETE of largest tumor	1.97 (0.78, 4.95)	0.151		
Multiplicity of tumor	0.89 (0.39, 2.03)	0.792	2.79 (0.3, 23.7)	0.347
Bilaterality of tumor	0.65 (0.28, 1.52)	0.323	0.03 (0.03, 2.3)	0.225
Laterality of LN[a]	1.02 (0.58, 1.78)	0.931	2.0 (0.7, 5.4)	0.171
LD of LN	1.08 (0.98, 1.2)	0.09		
SD of LN	1.34 (1.1, 1.64)	0.004	1.19 (0.9, 1.4)	0.085
L/S ratio of LN	0.30 (0.15, 0.63)	0.001	0.89 (0.2, 3.3)	0.87
Round shape (L/S <2.0)	3.56 (1.5, 8.5)	0.004	0.57 (0.1, 2.3)	0.432
Round shape (L/S <1.5)	5.02 (1.7, 15.1)	0.004	3.42 (0.9, 13.2)	0.075

Note-US, ultrasound; OR, odds ratio; ETE, extrathyroidal extension; LN, lymph node; LD, long diameter; SD, short diameter; long-to-short diameter ratio, L/S ratio

*[a]Contralateral location of the LN with respect to the primary tumor

Supplementary Table S3. Association of nodal size, shape parameters and primary tumor characteristics with malignant LNs in US probably benign LNs.

US Features	Univariable		Multivariable	
	**Crude OR (95% CI)**	***p***	**Adjusted OR (95% CI)**	***p***
Diffuse thyroid disease	2.25 (0.13, 37.5)	0.572		
Maximal diameter of largest tumor	1.04 (0.9, 1.2)	0.526		
Gross ETE of largest tumor	6.80 (0.39, 117.6)	0.187	9.1 (0.4, 203.4)	0.165
Multiplicity of tumor	2.71 (0.16, 45.4)	0.487	5.0 (0.2, 126.4)	0.328
Bilaterality of tumor	NA	0.998		
Laterality of LN[a]	0.3 (0.04, 2.8)	0.304	3.5 (0.17, 72.0)	0.417
LD of LN	1.05 (0.9, 1.26)	0.539		
SD of LN	1.13 (0.71, 1.8)	0.616		
L/S ratio of LN	1.14 (0.09, 14.1)	0.918		
Round shape (L/S <2.0)	5.627E-9	0.998		
Round shape (L/S <1.5)	NA	0.998		

Note-US, ultrasound; OR, odds ratio; ETE, extrathyroidal extension; LN, lymph node; LD, long diameter; SD, short diameter; long-to-short diameter ratio, L/S ratio

*[a]Contralateral location of the LN with respect to the primary tumor
